# The Effect of Ca^2+^ and Mg^2+^ Ions Loaded at Degradable PLA Membranes on the Proliferation and Osteoinduction of MSCs

**DOI:** 10.3390/polym14122422

**Published:** 2022-06-15

**Authors:** Sugoi Retegi-Carrión, Ana Ferrandez-Montero, Alvaro Eguiluz, Begoña Ferrari, Ander Abarrategi

**Affiliations:** 1 Center for Cooperative Research in Biomaterials (CIC biomaGUNE), Basque Research and Technology Alliance (BRTA), 20014 San Sebastian, Spain; sretegi@cicbiomagune.es; 2 Institute of Ceramic and Glass, Spanish National Research Council (CSIC), 28049 Madrid, Spain; alvaro.eguiluz@icv.csic.es; 3 Laboratoire de Physicochimie des Polymères et des Interfaces (LPPI), I-Mat, CY Cergy Paris Université, CEDEX, 95000 Neuville sur Oise, France; 4 Ikerbasque, Basque Foundation for Science, 48009 Bilbao, Spain

**Keywords:** biodegradable membranes, ion delivery system, osteogenic ions, colloidal processing, in vitro cell culture

## Abstract

Biodegradable membranes, including Polylactic acid (PLA)-based membranes, are commonly used in bone-tissue-related clinical procedures as biointerface to promote bone tissue regeneration. Calcium (Ca^2+^) and Magnesium (Mg^2+^) ions have been related to the promotion of osteogenesis, where the PLA membranes could be used as carrier and delivery substrate for them to provide osteogenic properties to this material. For this aim, a new ion delivery system based on biodegradable PLA membranes loaded with Mg and hydroxyapatite (HA) particles has been processed by the combination of tape casting and colloidal route. Materials characterization shows that the incorporation of Mg and HA particles changes the surface and hydrophobicity of the PLA membrane, and the in vitro degradation test shows Mg^2+^ and Ca^2+^ ion release and occasionally the precipitation of different ion species onto the membrane surface. Mouse and human Mesenchymal Stem Cells (MSC) were used to define the biocompatibility and bioactivity of these PLA membrane composites, and data indicated Mg^2+^ promotes cell proliferation and potentiates osteoinductive signals, while Ca^2+^ induces the expression of ALP osteogenic marker in human MSCs. Biodegradable PLA membranes loaded with Mg and HA particles is a promising new ion delivery system of Mg^2+^ and Ca^2+^ ions that provides osteogenic signals and works as functional biointerface interfaces with bone tissues.

## 1. Introduction

Bone defects caused by trauma, infection, tumors or inherent genetic disorders, together with the general aging of the world population, still remain a significant clinical challenge that usually requires surgery and implantation of **bone grafting materials**. Unfortunately, some current treatments present limited effectiveness [1,2]. While bone grafting is usually related to 3D structures, membranes are indeed commonly used to guide, help and promote bone tissue regeneration in clinical settings. For example, guided bone regeneration (GBR) is a standard clinical procedure used to treat maxillofacial bone defects, and it is based on implantation of barrier membranes protecting the bone-to-be area and promoting regeneration [3]. In this context, the development of new bone regenerative materials is the driving force to seek new strategies aimed at improving patients’ comfort as well as improving the surgical procedures.

It is widely accepted that an ideal **bone implantable compound** should mimic tissue’s properties and to achieve that, the mainstream is the generation of composites of different materials [4]. Following this strategy, the resulting implant can meet the required mechanical properties, as well as the osteoconductive, osteoinductive and preferably biodegradable properties required for the regeneration of the bone. In this sense, biodegradable and bioabsorbable temporary implants doped with bioactive phases are gaining popularity in multiple bone-healing applications. The basic aim pursued at generating these composites is achieving tissue regeneration towards new bone formation and integration, with complete final degradation of the implanted compound [5,6]. Polylactic Acid (PLA) is a biodegradable material that can serve as a scaffold, and PLA-based composites are useful as temporary implants and membranes for osteosynthesis applications [7,8,9,10,11]. Specifically, PLA has gained much attention as a GBR membrane because of its handleability and biodegradability, and it has demonstrated that it is effective in this application of bone defects, favoring bone formation [12].

**Bioactive ions** with alleged osteogenic properties are gaining interest in the field. It is known that some ions are essential for the development and regeneration of bone tissue, while ions such as Ca^2+^, Cu^2+^, Sr^2+^, Mg^2+^, Zn^2+^, and B^3+^ amongst others, have proven activity in terms of enhancing osteogenesis [13]. Biodegradable materials play a crucial role in the possible application of these ions for bone promotion, as they serve as a carrier and release substrate, thereby modulating the ions’ osteogenic properties. The biodegradable materials used for ions delivery have been assayed in multiple morphologies as scaffolds [14,15], hydrogels [16], micro and nanoparticles [17] and membranes [18], among others.

Bioceramic materials based on **Calcium (Ca)**, as tricalcium phosphate (TCP) or specially hydroxyapatite (HA) are similar to bone inorganic fraction and therefore they have been used as inorganic load in PLA-based composites [2,19,20,21,22]. Regarding PLA/HA composites, improved mechanical properties have been reported due to the incorporation of HA into PLA, including increased elastic modulus and compressive strength [23,24]. The addition of HA also stimulates cell adhesion and growth, with minimal inflammatory response in in vivo implantation procedures [25]. Moreover, further incorporation of additional organic phases to these composites, such as chitosan, may result in improved bone cell behavior on PLA composite surfaces [26]. Additionally, HA or HA-based composites have been widely used as GBR membranes [27].

In parallel to ceramics, other inorganic compounds have been used in combination with PLA to provide additional properties. For example, **Magnesium (Mg)** is a metal suggested as an osteoinductive biometal due to its multiple roles in bone physiology, including roles in bone formation, growth and mineralization processes [28]. Mg alloys have been studied as a promising alternative membrane for GBR procedure [29]. As a shortcoming, Mg possesses strong reactivity, resulting in local alkalization and hydrogen gas formation close to the implantation area, which hinders its applicability in bone-regeneration context [30]. In PLA/Mg composites assayed for osteosynthesis [31,32,33], PLA has the ability to stabilize the reactivity and fast biodegradability of Mg [34], while Mg and its media alkalinization neutralize the acidification that generally occurs due to the degradation of the PLA polymer. In regard to the neat polymer, Mg enhances its mechanical properties, particularly the creep strength and compression modulus [35], and according to in vitro tests, the PLA/Mg composites are totally biocompatible and produce an increase of the cell viability and proliferation [36].

The combination of Mg^2+^ and Ca^2+^ in composite biomaterials has been reported previously in magnesium-substituted hydroxyapatite [37,38,39], Mg bioglass [40] or Mg/HA composites [41]. Some works have demonstrated the potential of Mg^2+^ and Ca^2+^ in GBR membranes thanks to their bioactivity and biodegradability [42]. Mg/HA composites and their impressive biocompatibility, biodegradability and the possibility to modify the composition ratios has led to further interest in research. The **synergism of Mg/HA** has been studied for the field of orthopedic fracture fixation [41], where HA-based coatings on Mg alloys substrates lead to better biodegradation modulation and improve in vitro osteoblast adherence and proliferation [43,44]. However, biodegradable polymer composites with HA/Mg filler have not been studied yet.

**Colloidal processing techniques** are well known in biomaterials, and expressly in composites, due to the extraordinary effectivity of the wet mixture of different species, polymers and inorganic particles, in the homogeneity of the final microstructure [36,45]. Tape casting of colloidal suspension results in membrane formation with successful homogeneity and dispersion of the incorporated microparticles reported in the literature [46]. Moreover, this shaping technology allows modulating the amount of inorganic particles in the composite membrane, providing versatility to process specific composites.

The incorporation of HA and Mg particles to PLA by colloidal suspension and further tape casting would yield composites with improved degradability and osteoinductive properties. In order to prove that, first we implemented the composite preparation method. Once membranes were generated, we tested their properties, including cell adhesion, proliferation and osteoinduction ability on human and mouse Mesenchymal Stem Cells and we made a relation of these results with the presence of Mg and HA filler and the release of Mg^2+^ and Ca^2+^ ions. Results indicate that the PLA/Mg/HA ion delivery system based on PLA membranes is a feasible and promising approach to provide osteogenic signals to clinically useful implantable materials.

## 2. Materials and Methods

### 2.1. Processing of PLA Composites and Material Characterization

Mg particles (Mg > 99.8%, Nitroparis, Spain) and HA particles (Ca:P = 1.67, Plasma Biotal Limited, Derbyshire, UK) of less than 50 µm (d_V50_ = 29.22 µm) and 3 µm (d_V50_ = 2.76 µm) in diameter, respectively, were used. PLA solutions were prepared by dissolving pellets of PLA polymer 2003D with D-isomer content of 4.25% (Natureworks^®^, Minnetonka, MN, USA) in Tetrahydrofuran (THF, Panreac, Darmstadt, Germany). Mg and HA modified particles were mixed with the PLA solution under vigorous mechanical stirring. The composite membranes were prepared by the tape casting technique. Experimental details were provided elsewhere [36].

The surface of inorganic particles was previously modified in aqueous suspension by adsorbing the exact amount of a cationic polyelectrolyte as PEI (Polyethylenimine Mv 25,000, Sigma Aldrich, Taufkirchen, Germany) needed to preserve their chemical and colloidal stability. Mg aqueous suspension was prepared in deionized water by mechanical stirring, adjusting the pH value over 12 with TMAH (Tetramethylammonium hydroxide) and adding 0.2 wt.% of PEI on the basis of the Mg content. After that the suspension was milled for 45 min with nylon balls to fully disperse and stabilize the Mg particles by breaking soft agglomerates and adsorbing PEI [47]. Similarly, HA colloidal suspensions were also formulated in deionized water at pH 8 and 1 wt.% of PEI, and milling for 60 min with zirconia balls [48].

The PLA/Mg PLA/HA or PLA/Mg/HA suspensions were cast on a glass plate using a moving tape casting device with two doctor blades. The casting parameters were 10 mm s^−1^ of casting velocity and 100 μm of gap height between the blades and the carrier tape. After drying in air at room temperature overnight, the membranes were further dried at 60 °C for 24 h. Membrane compositions are summarized in Table 1.

### 2.2. In Vitro Characterization of the Membranes Properties

**Figure 1:**
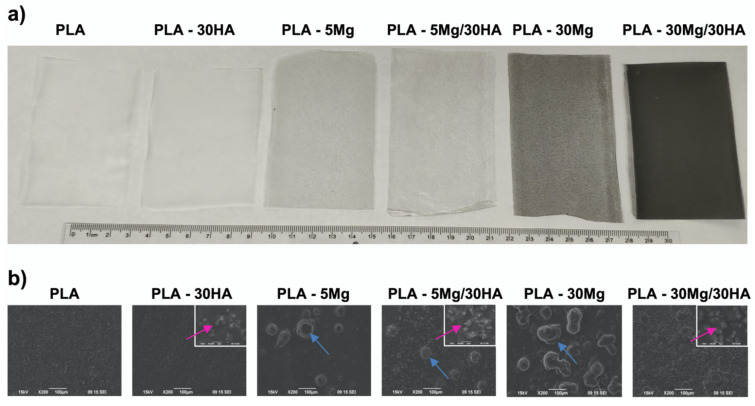
(**a**) **Membranes macroscopic appearance and** (**b**) **SEM images of the different membranes**. (**b**) Blue arrows indicate location of the Mg particles observed as structures of 50 to 120 µm, while pink arrows highlight HA particles of 1 to 3 µm.

The roughness images of the samples were studied by an optical profilometer Zeta (Zeta Systems, Hallbergmoos, Germany) and analyzed by the program Zeta (KLA-Tenco). Main roughness parameters were evaluated: the superficial average roughness, S_a_, the average height to the peaks, R_h_, and the S_ku_, kurtosis parameter. The membrane surfaces were sputtered (gold/palladium, Alto 1000, Galan) and observed by scanning electron microscopy (SEM) (JEOL JSM-6490 LV at 15 kV (Figure 1 and Supplementary Figure S1), or, FEG Hitachi S4800 at 15 kV equipped with energy dispersive X-ray spectroscopy (EDS) and INCA energy software (Oxford Instrument, Abingdon, UK).

To compare the handleability of ion-doped PLA membranes, a video was recorded manipulating different membranes, including Bio-gide collagen membrane (Geistlich, Schlieren, Switzerland) that was used as a standard of clinically used membranes for GBR (Supplementary Video S1).

In vitro degradation of the composites was studied by measuring ions’ release, water uptake and weight loss of the membranes during 20 days of immersion in Phosphate Buffered Saline solution (PBS). The PBS medium used do not content Mg^2+^ and Ca^2+^ ions in order to avoid any interference with the ions release measurement. Inductively coupled plasma–optical emission spectroscopy (ICP-OES, IRIS ADVANTAGE, Termo Jarrel Ash) was used to determine the concentration of Mg^2+^ and Ca^2+^ in the immersion medium. Samples of 5 cm^2^ were immersed in 25 mL of PBS, and an aliquot of 2 mL was removed each time we measured the ions release. After each extraction, 2 mL of fresh PBS solution was added to maintain degradation conditions. Simultaneously, other batches of samples were prepared under similar degradation conditions to determine the weight loss and the water uptake by each composition. For each degradation time, the mass of wet membranes was determined immediately after removing from the PBS solution, while the mass loss was measured after samples dried overnight at 60 °C. Each time point is represented as the average of the triplicate test per specimen. The error of the measurement was less than 4%, which was not represented in the release experiment in order to present the result clearly.

The contact angle measurement of all the samples and the control was carried out using Drop Shape Analyzer—DSA 100 (Krüs Scientific, Toulouse, France). Sessile drop method was exploited to calculate the contact angle after 10 s; a 2 µL nanopure water drop was dispensed at 2.67 µL/sec. The fitting method was Ellipse (Tangent-1) and the baseline was selected manually. At least six measurements on different areas of each membrane specimen and glass controls were averaged.

### 2.3. In Vitro Cell Culture Studies

In order to perform cell culture studies, C3H/10T1/2, Clone 8 (ATCC^®^ CCL-226™) mouse embryonic Mesenchymal Stem Cells [49] and Human Bone Marrow-Derived Mesenchymal Stem Cells (LONZA PT-2501) were cultured and incubated under standard conditions of 37 °C and 5% CO_2_ in Basal Medium Eagle (BME; 41010-026; Gibco, New York, NY, USA), MEM Alpha (αMEM; A10490-01; Gibco, New York, NY, USA) and Mesenchymal Stem Cell Basal Medium (MSCBM; PT-3238; Lonza, Basel, Switzerland), respectively, both supplemented with 10% fetal bovine serum (10500-064, Gibco) and 1% antibiotics (100 U mL^−1^ penicillin, 100 μg mL^−1^ streptomycin; Gibco).

For experimental in vitro cell culture, membranes were first sterilized using UV radiation for 30 min and then handled in sterile conditions. Pieces were taken using a disposable sterile 5 mm in diameter biopsy punch (94158BPP-40F; Kai Medical, Seki, Japan) and placed in 48-well plate (Corning^®^ Costar^®^ TC-Treated Multiple 48 Well Plates; CLS3548-100EA; Merck, Taufkirchen, Germany). Then, a drop of media with 5 × 10^5^ cells was carefully deposited on top of each membrane, ensuring the drop would be stable on the membrane surface. After 4 h, once cell attachment to material was obtained, 500 µL of media was added to each well. Further assessment confirmed there was no leakage of adherent cells from PLA surfaces to well surface, while specimens were transferred from their well for further studies.

Cell adhesion and spreading on membranes were observed by Actin and DAPI staining. Briefly, samples were fixed with formalin solution neutral buffered, 10% (HT501128; Sigma Taufkirchen, Germany). For cell visualization, actin filaments were stained with ActinRed^TM^ 555 ReadyProbesTM reagent (R37112, Invitrogen, Waltham, MA, USA) for 30 min and DAPI staining (dilution 1:2000 in PBS) was used as a counterstaining to analyze the nucleus. Samples were transferred to histology slides and visualized in a Carl-Zeiss LSM 510 confocal microscope (objective: Plan Neofluar 20x; NA 0.5; Air; Working distance 0.61 mm). Image processing and analysis were conducted using Zen 2012 (Blue Edition) Software and ImageJ.

For measuring cell proliferation, Deep Blue Cell Viability Kit (424701; BioLegend, San Diego, CA, USA) reagent at a 10% of the total medium was added to each testing well. Samples were incubated at 37 °C for 150 min. Then, the medium was transferred to a black 96-well plate and fluorescence measurement was performed with an excitation wavelength of 535 nm and an emission wavelength of 590 nm (Tecan GENiosTM plate reader). Blank readouts were subtracted.

For inducing cell differentiation, hMSCs were cultured in MesenCultTM Osteogenic Differentiation Basal Medium (05466; StemCell, Vancouver, BC, Canada). C3H/10T1/2 cell differentiation was induced with growing media supplemented with 50 µg mL^−1^ of L-ascorbic acid (A5960, Sigma-Aldrich, Taufkirchen, Germany) and 2 mM of β-glycerophosphate (G9422, Sigma-Aldrich). After 7 days, Alkaline Phosphatase Activity was measured using StemTAG^TM^ Alkaline Phosphatase Activity Assay Kit (CBA-301, Cell Biolabs, Madrid, Spain) and following the indications of the manufacturer, including Alkaline Phosphatase Activity data normalization to cell proliferation data.

In this work, data are shown as ±SD from at least 3 different independent experiments. For statistical analyses, ANOVA was performed using Graphpad Prism software. The level of significance was set at 0.05. (ns *p* > 0.05; * *p* < 0.05; ** *p* < 0.01; *** *p* < 0.001, **** *p* < 0.0001).

## 3. Results and Discussion

### 3.1. Delivery of Mg^2+^ and Ca^2+^ Ions Facilitates Water Uptake Thereby Modulating the Membrane Degradation

The colloidal method used to form PLA membranes allows the loading of a high concentration of Mg and HA in them. Therefore, previous experience was used to decide the loading ranges to be used in this study. Regarding Mg, Mg-based materials readily oxidize in the presence of water due to their low thermodynamic stability, liberating hydrogen gas [30]. The tolerated amount of hydrogen by the human body is 2.25 mL/cm^2^/day, so in terms of hydrogen accumulation, membranes should not exceed this limit to be suitable for biomedical use [50]. In a previous work, PLA/Mg membranes with filler concentrations up to 50 wt.% (PLA-50 Mg) were tested, evidencing the fast Mg corrosion and simultaneous ions and hydrogen liberation during the first two weeks of the immersion in PBS. In that work, 30 wt.% of Mg (PLA-30 Mg) was considered the maximum Mg loading with a safe limit for the hydrogen liberation [36]. Moreover, previous macromechanical characterization of the PLA/Mg-membranes in terms of stiffness by Dynamic Mechanical Thermal Analysis demonstrated that the addition of Mg modifies the Young Modulus of the membranes, from 2471 MPa for PLA membranes to 2528 MPa for 10 wt.% of Mg (PLA-10 Mg), 2108 MPa for PLA-30 Mg and 1834 MPa for PLA-50 Mg. This indicates that the loading of a high amount of Mg may drastically modify the elasticity of the membranes. Thus, we first compared the handleability of doped PLA membranes with commercially available clinically used membranes and proved it was similar to them (Supplementary Video S1). Based on these results, a compromise between bioactivity and macromechanical properties leads to the exclusion of highly Mg loaded membranes from this study, considering PLA-5 Mg, PLA-10 Mg and PLA-30 Mg as testable materials.

Compared with Mg particles, HA particles show low reactivity. Once incorporated in the PLA membranes by the colloidal methodology, HA particles also show lower particle size and higher dispersion than Mg particles, resulting in an elevated surface exposure (Figure 1). Moreover, preliminary data indicate the addition of HA particles showed no detrimental effect in PLA membrane properties. Therefore, the range of HA considered to be incorporated to PLA membranes was higher than that selected for Mg, but still we kept both particle concentrations in the same range, aiming to be able to compare further effects. Therefore, we tested 10 wt.% of HA (PLA-10 HA), 30 wt.% of HA (PLA-30 HA) and 50 wt.% of HA (PLA-50 HA).

Once the starting concentrations were selected, ions release, water uptake and membrane degradation were determined for membranes whose compositions are described in Table 1. PLA is a long-term degradable material, and therefore we tested at 30 days knowing that at this time point PLA membranes should remain stable. Moreover, we tested in watertight conditions to simulate the cell culture test setting. The pH value of the PBS solution during the ions release experiment was also monitored, and in no case was the buffer capacity of PBS exceeded. For this reason, pH evolution is not shown in this study. This phenomenon was already reported in previous works of in vitro degradation of PLA/Mg and HA composites [51].

**Figure 2:**
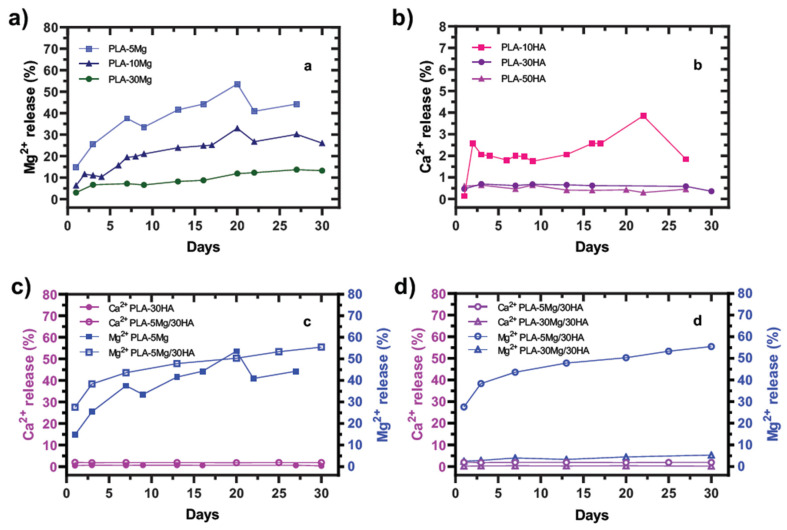
**Evolution of Mg^2+^ and Ca^2+^ ions release at immersion in PBS.** Data are shown normalized by the amount of Mg and Ca incorporated to the composites. (**a**) Mg^2+^ release in PLA-5 Mg, PLA-10 Mg and PLA-30 Mg composites. (**b**) Ca^2+^ release at PLA-10 HA, PLA-30 HA and PLA-50 HA composites. (**c**,**d**) Comparative evolution of Mg^2+^ and Ca^2+^ ions release in mono- and biphase- composites. (**c**) PLA-30 HA, PLA-5 Mg, and their biphase composite. (**d**) Release at PLA-5 Mg/30 HA and PLA-30 Mg/30 HA.

Mg^2+^ and Ca^2+^ release is shown as a percent normalized by the real amount of Mg and Ca of in the tested sample. These values were plotted in Figure 2a,b respectively. Absolute data are provided in Supplementary Figure S2. Figure 2a,b shows that the higher the content of Mg or HA is in the membrane, the lower the relative amount of Mg^2+^ and Ca^2+^ ions release is. This fact is related to the solubility limitation of the Mg and HA species and the dissolution and re-precipitation processes taking place simultaneously.

Mg^2+^ ions release is due to the spontaneous redox reaction between this alkaline metal and the PBS water solution, producing Mg(OH)_2_ which is soluble in PBS medium. In absolute lixiviation values, the Mg^2+^ release increases with the Mg content of the membrane. 30 mg/L of Mg^2+^ ions was released for PLA-30 Mg samples after 7 days, while the PLA-10 Mg and PLA-5 Mg samples liberated around 19–17 mg/L of Mg^2+^. In both cases, release data tend to stabilize later on but continue increasing within the 30 days of immersion, while in any case the data are higher than the water solubility of Mg(OH)_2_, suggesting oversaturation in the media and possible re-precipitation.

The behavior of the Ca^2+^ ions lixiviation differed qualitatively and quantitatively from the Mg^2+^ releases. The solubility of HA is very low compared to Mg(OH)_2_, and it is well described that for HA saturated solutions the equilibrium is achieved with a surface-precipitated layer [52]. In our experiments, the amount of liberated Ca^2+^ ions was limited to 2–3 mg/L, which is higher than the reported solubility of HA [52]. That means that the HA was soluble in PBS medium while the trend of Ca^2+^ release stabilized after a couple of days of immersion and then remained constant in saturation in all cases. In fact, despite a strong release of Ca^2+^ registered during the first 14 days of immersion in PLA-50 HA composites (overpassing 3 mg/L and data corresponding to the lixiviation of only a 0.64% of Ca in the membrane), the cumulative concentration of Ca^2+^ ions stabilizes at similar values to those of the PLA-10 HA and PLA-30 HA membranes, decreasing down to 1.5 mg/L after 30 days of immersion, due to HA re-precipitation. In this case, the effectiveness of Ca^2+^ release at the membrane surface is also limited by its solubility, demonstrating that in the PLA-50 HA membranes, the HA particles content is in excess, remaining inside the membrane without any effect over the surface phenomena.

Consequently, for further studies of hybrid materials with Mg and HA particles, the HA content was fixed to 30 wt.% (PLA-30 HA) varying the Mg content from 5 to 30 wt.% (PLA-5 Mg to PLA-30 Mg). First, we studied the release behavior of Ca^2+^ ions as a function of the presence of a fixed content of the Mg reactive metal. Plot in Figure 2c shows both ions released in PLA-5 Mg, PLA-30 HA and PLA-5 Mg/30 HA membranes. It seems both Mg^2+^ liberation and Ca^2+^ lixiviation could have a synergism effect in the membrane containing both bioactive particles, since ions release is higher in the PLA-5 Mg/30 HA membrane than in mono-biophase composites. The Ca^2+^ release stepped up with the presence of Mg as a consequence of the fast degradation of the membrane provoked by the catalyst, Mg particles. The presence of both bioactive particles increases the water permeability and accelerates the degradation of composite. The liberation of both ions quickly stabilizes and maintains the liberation in a 2% and 50% of the content of Ca^2+^ and Mg^2+^ ions in the bioactive particles of the membrane, HA and Mg, respectively. However, plot in Figure 2d shows that the further addition of more Mg particles in PLA membranes strongly decreases the Ca^2+^ ions release. In fact, the Ca^2+^ lixiviation decreases one order of magnitude in absolute values, from 2 g/L to 0.2 g/L when the content of Mg in the membrane changes from 5 (PLA-5 Mg/30 HA) to 30 wt.% (PLA-30 Mg/30 HA). Assuming that a higher amount of Mg in the membrane induces higher degradation, we concluded that the saturation of the medium by Mg dissolution under watertight conditions is the factor inhibiting the Ca^2+^ lixiviation in these high Mg carrier PLA membranes. Oppositely, Ca^2+^ lixiviation seems to have no effect on the Mg^2+^ release.

**Figure 3:**
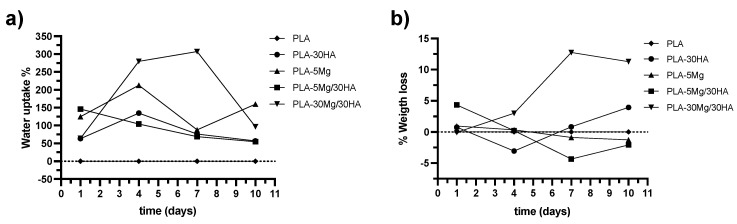
(**a**) **Water uptake evolution and** (**b**) **weight loss trend.** Data were obtained at different immersion times in PBS. Tested samples were PLA; mono-biophase composites PLA-30 HA and PLA-5 Mg; bi-phases composites PLA-5 Mg/30 HA and PLA-30 Mg/30 HA.

During release assays, the degradation of the Mg carrier PLA membranes was evident, including apparent membrane hydration. As there is no degradation or water uptake in PLA membranes just by immersion in PBS, these parameters were analyzed during the first 10 days to better understand the dissolution-precipitation process in Mg, HA and hybrid ion carrier membranes. Evolution of both parameters is plotted in Figure 3a,b, respectively. In the case of water uptake behavior, all ion carrier samples hydrated and the sample that adsorbed the larger amount of water was the composite PLA-30 Mg/30 HA. This could be associated with the high amount of hydrophilic inorganic phases (>35 vol.% of inorganic particles) in this composite, which enhances the swelling. At the PLA-5 Mg/30 HA composite, the water uptake decreases over time because the lower content of inorganic and hydrophilic phases decreases the swelling effect, evidencing the membrane degradation. This similarly occurs for PLA-5 Mg and PLA-30 HA samples used as references.

A continuous weight loss trend is evident for PLA-5 Mg and PLA-5 Mg/30 HA membranes related to the material degradation. However, weight gain was registered for PLA-30 Mg/30 HA, PLA-30 HA composites, a phenomenon that cannot be attributed to water uptake, as samples were dried before weighing them. Therefore, we consider that it is related to the reprecipitation of the released ions in different species as HA and/or Mg(OH)_2_ onto the membranes’ surface. Weight gain due to precipitation is more evident for the PLA-30 Mg/30 HA material, since it is the highest inorganic content, but it is also appreciable for PLA-30 HA after 4 days of immersion and for PLA-5 Mg/30 HA after 7 days of immersion.

### 3.2. Mg and HA Particles Modulate the Surface Properties of the PLA Membranes

Surface properties are thoroughly related to materials biocompatibility, as cells adhere and proliferate on it. The ion delivery and hydration studies performed in PBS media suggest different surface phenomena are taking place in ion carrier PLA membrane surfaces, including surface degradation and crystal structure precipitation on it. Therefore, we decided to study surface properties obtained after those processes.

**Figure 4:**
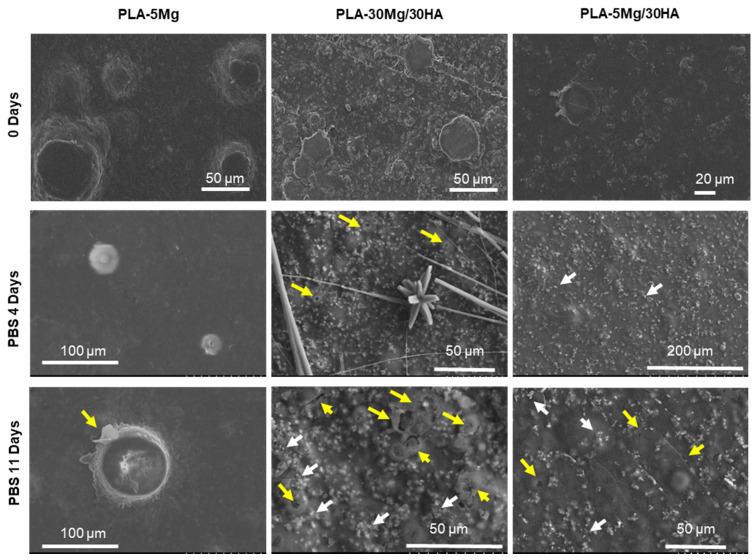
**SEM micrographs of the surface of the membranes after immersion in PBS.** Yellow arrows indicate PLA surface degradation and white arrows indicate precipitated Ca nodules.

SEM images in Figure 4 show the evolution of different PLA composites after immersion in PBS. Previous characterization demonstrates that Mg particles can be observed in the surface as fully surrounded by PLA in these membranes [36]. After 4 days of immersion, some Mg particles were already degraded and membranes exhibited small pits at the surface near the Mg particles. Longer times of immersion (11 days) promote the further Mg dissolution and the continued membrane degradation breaking PLA surface (yellow arrows in the images). Regarding HA particles, they can easily be observed in the surface, and after immersion Ca deposits are formed (white arrows in the images).

Micrographs of PLA-30 Mg/30 HA membranes after immersion (Figure 4) exhibit main effects of degradation: the precipitation of Mg(OH)_2_ forming flowerlike and needle-like structures (See day 4 at Figure 4), a cracked/porous polymeric matrix and the craters formed by the H_2_ release due to the redox reaction of Mg dissolution (yellow arrows), and the HA particles precipitated onto the membrane surface (white arrows). Consequently, results prove that the high reactivity of Mg steps up the membrane degradation and the precipitation of HA and Mg(OH)_2_ species at the membrane surface than could cause difficult cell proliferation. These data were highly illustrative of all the dissolution-precipitation processes related to Mg and HA components and taking place after immersion in PBS, while they indicated that any composition with a high amount of Mg (PLA-30 Mg; PLA-30 Mg/30 HA) was not suitable for further in vitro cell culture testing in watertight conditions.

The combination of 30 wt.% of HA and 5 wt.% of Mg in the composite (PLA-5 Mg/30 HA) seems to be optimum in terms of the liberation of osteoinductive ions with moderate membrane degradation. As shown in Figure 2, lixiviation of Ca^2+^ promotes a constant concentration of these ions over the saturation limit in the immersion media, also maintaining HA dissolution-precipitation reactions. Moreover, a constant and moderate Mg^2+^ release assures the suitable membrane degradation during cell culture, avoiding fast H_2_ release or the Mg(OH)_2_ precipitates’ formation. Regarding membrane degradation, Figure 4 shows that although Mg^2+^ and Ca^2+^ ions are released, the membrane surface preserves its features during the first 4 days of immersion. Only small agglomerates of HA precipitates are observed. Further immersion times show moderate PLA surface degradation compared to composites with a high amount of Mg.

Aiming to better define the biocompatibility of PLA composites at cell-seeding moment, surface roughness and the water contact angle measurements at as-shaped membranes (before immersion in PBS) were performed, as those properties are also related to the cell-behavior on the seeded surface. The values of main parameters are collected in Table 2.

**Figure 5:**
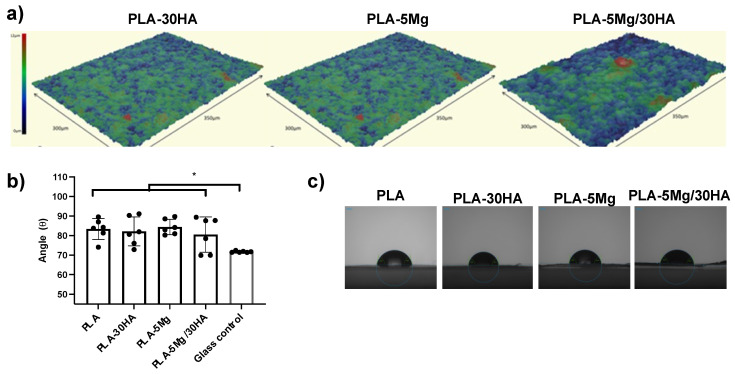
**Membranes surface properties.** (**a**) Topography images of the membrane surface of the PLA composites. (**b**) Water contact angle measurements performed on different surfaces and (**c**) representative pictures (*n* = 6 replica per surface).

Table 2 collects the characteristic parameters defining the roughness of the membrane surfaces and also illustrated in Figure 5a–c, which shows the images of the topography of the composite membranes. The average roughness of the composite, S_a_, is associated with the filler loading. The greater the particles content is, the higher the number of irregularities is on the membrane surface. The average roughness of composite membranes is around 1.1–2.3 µm. The average height, Rh, is mainly determined by the surface effect of the size of the dispersed particles in the membrane, and the Kurtosis factor, S_ku_, is a measure of the sharpness of the roughness profile and depends on the number of particles and the distance between them. The presence of Mg particles, 20–50 µm in size, increases the average height to 4.1 µm in PLA-5 Mg and 5.9 µm in the composite with two fillers PLA-5 Mg/30 HA, while the incorporation of HA particles reduces the Kurtosis factor one order of magnitude as corresponding with the particle size of the ceramic filler (1–3 µm) and the higher amount of HA particles in the tested membrane. This indicates that, although the roughness is sharper in the membranes with Mg, the presence of a high number of HA particles reduces the profile of the roughness, leading to a less abrupt surface in the bi-phases composite. Contact angle data in Table 2 and Figure 5 suggest no effect of the hydrophilic nature of the inorganic particles in this parameter [53,54].

### 3.3. Mg and HA Particles Incorporation on PLA Membranes Improves Cell Biocompatibility and Osteogenic Differentiation Ability of Mesenchymal Stem Cells

Materials surface characterization study indicated an initial hydrophobic surface in all membranes (Figure 5), while PBS immersion indicated hydration and sustained ion delivery (Figure 2 and Figure 3) triggering surface degradation during the time (Figure 4). The incorporation of 50 wt.% of HA (PLA-50 HA) did not improve HA delivery, while the incorporation of 30 wt.% of Mg (PLA-30 Mg) induced too-fast surface degradation. With these premises, we decided to define cell culture analysis in selected membranes, which had 30 wt.% of HA (PLA-30 HA) and 5 wt.% of Mg (PLA-5 Mg), individually and in combination.

For cell culture studies, samples were cut in round disks and transferred to cell culture multiwell plates. PLA disks were able to allocate up to 30 µL of cell culture media and remained submerged on the bottom once the multiwell plate was filled with culture media, indicating they were useful as flat surface for cell seeding purposes, and still removable from the well and transferable to other plate for further studies (Supplementary Figure S3). As the cell culture control surface, optical grade round glass coverslips were used, which were also removable from the seeding well. As cell culture medium present different amounts of Ca and Mg inorganic salts, cells grown in control and PLA surfaces are especially needed with comparative purposes in order to accurately define the effect of Ca and Mg inorganic salts locally delivered at cell-adhesion surface microenvironment.

Multilineage progenitor cells are those considered to be responsible for bone tissue regeneration. Therefore, two different cells were used to test the bioactivity of these membranes, both with Mesenchymal Stem Cell phenotype but different in terms of tissue-origin, cell-size and proliferation kinetics. Mouse embryo-derived C3H10T1/2 is a cell line with high proliferation rate and considered multipotent mesenchymal stem cell due to its ability to differentiate into adipose, osteo and chondral phenotype [55,56]. Primary cultures of human bone marrow-derived mesenchymal stem cells (hMSC) with proven multilineage differentiation ability to adipose, osteo and chondral phenotype are thoroughly used as translational cell-model in biomaterial testing studies.

**Figure 6:**
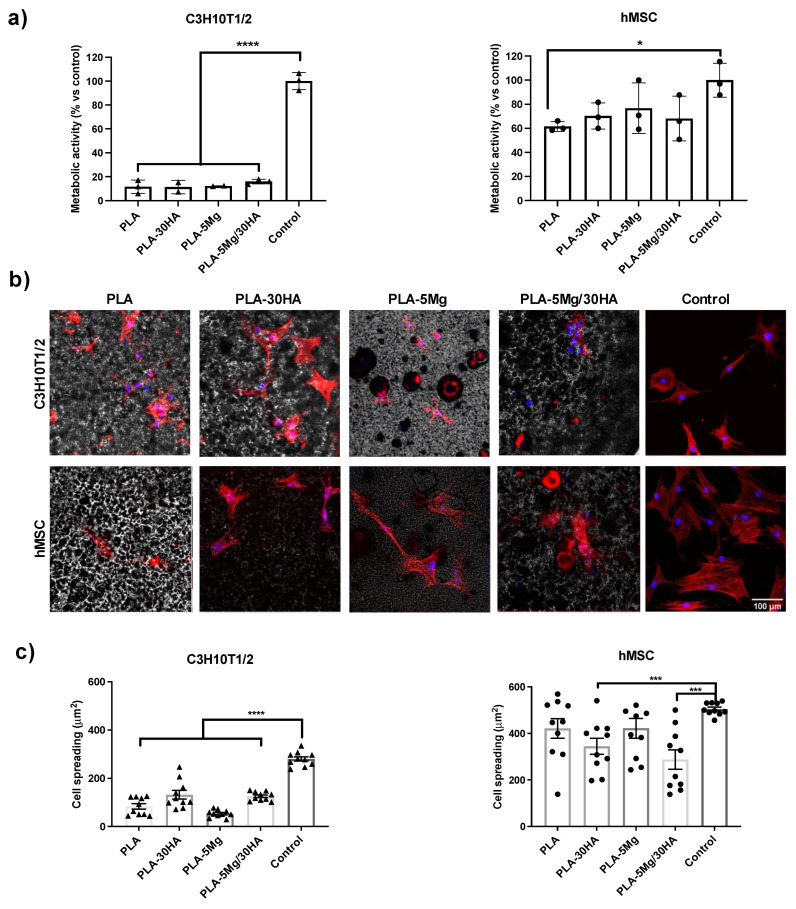
**Cell adhesion 24 h after cell-seeding**. (**a**) Metabolic activity measured with Alamar blue assay (*n* = 3 replica per surface). (**b**) Confocal images of cells on the different surfaces after staining for Actin cytoskeleton (Red) and DAPI nuclear staining (Blue) (scale 100 µm). (**c**) Quantification of cell shape and spreading on different surfaces (*n* = 10 cell per condition).

Taking into account the hydrophobic nature of PLA membranes and the effect of Mg and HA on surface roughness, we first tested cell adhesion to the selected membranes aiming to define the role of PLA, Mg and HA in initial biocompatibility of the membranes. Therefore, cells were seeded on membranes and tested at 24 h assuming from previous data that there was no significant degradation at that time point in membranes with the tested composition (Figure 6). Quantitative metabolic data and cell spreading and size measurements indicate statistical differences related to membrane composition and data were highly dependent on tested cell type.

C3H10T1/2 cells show low metabolic data in all PLA membranes (Figure 6a), suggesting lower cell adhesion on that surface compared to the control one, a behavior surely driven exclusively by cell response to the hydrophobic nature of PLA surface and not by the ions. Actin cytoskeleton staining was performed at this time point (Figure 6b), and C3H10T1/2 cells showed reduced cell spreading with more rounded shape and with less filopodia compared to cells seeded in the control surface. Occasionally, they were observed forming cell colonies or patches suggesting cell–cell interaction was stronger than the adhesion to the surface. Images revealed slightly better spreading on HA carrier surfaces, still far from the shape of cells seeded in the control surface. Although the cell spreading seemed slightly worse in Mg carrier surfaces’ a trend of cells to interact with Mg particles was observed. Quantitative data of cell spreading (Figure 6c) indicated C3H10T1/2 cells were smaller on all PLA surfaces with some differences related to ion cargo. In sum, for C3H10T1/2 cells, hydrophobicity of the PLA surface was the driver of cell adhesion and no effect was observed related to the presence of HA or Mg at this initial time point.

Quantitative metabolic data for hMSC cells (Figure 6a) indicated hMSCs seeded in HA and/or Mg-carrier PLA membranes showed similar metabolic data compared to control surface, suggesting a positive effect of both ions on these cells’ adhesion-seeding. Regarding hMSC cell spread, it turned out that it was very heterogeneous on all PLA surfaces compared to the control surface (Figure 6b,c). We consider this behavior in cell shape can be related to cell-material interaction but not specifically to the changes in the surface roughness observed in HA and Mg carrier membranes, as cell shape heterogeneity is the same for all PLA surfaces. In Mg and HA/Mg carrier surfaces, hMSCs cells were observed in close contact with Mg particles, but also in other areas of these surfaces, indicating that cells were not repelled by the presence of Mg in the surface, but at the same time they were not specifically attracted to adhere or migrate to Mg particles.

**Figure 7:**
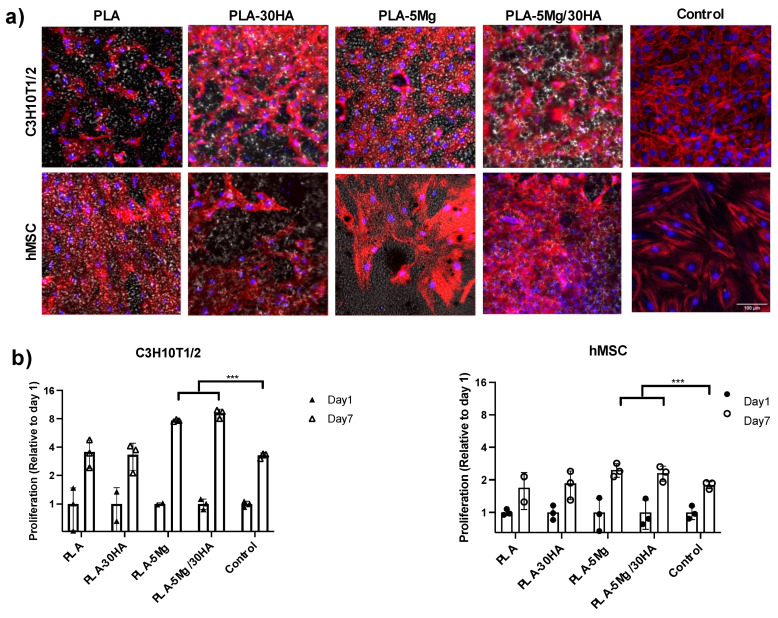
**Cell proliferation 7 days after cell-seeding.** (**a**) Confocal images of cells on the different surfaces after staining for Actin cytoskeleton (Red) and DAPI nuclear staining (Blue) (scale 100 µm). (**b**) Alamar blue cell metabolism assay data normalized to data obtained at 24 h to represent the increase associated with cell proliferation on each specific surface (*n* = 3 replica per surface).

**Figure 8:**
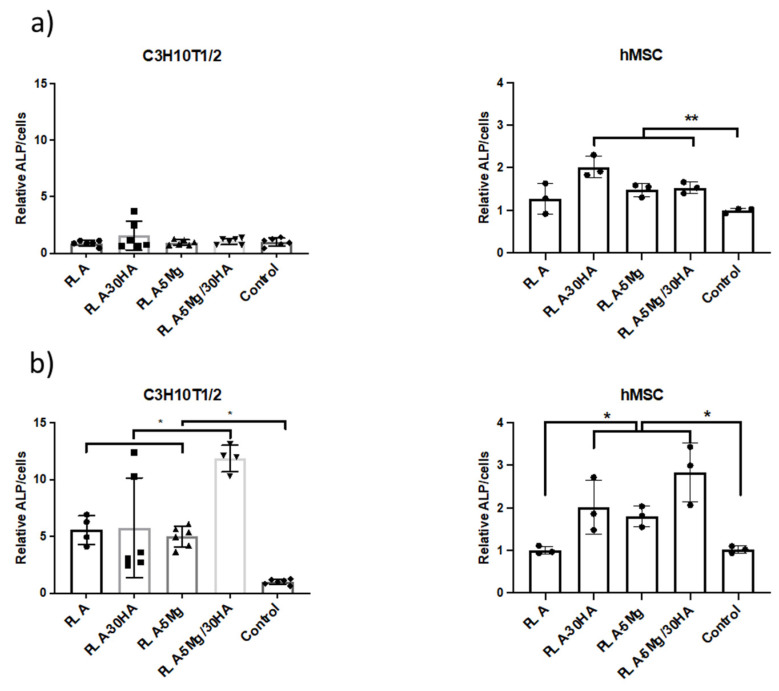
**ALP osteogenic differentiation marker activity measurement 7 days after cell-seeding**. ALP measurement after 7-day cell culture in normal media (**a**). ALP measurement after 7-day induction of cell-differentiation with osteogenic media (**b**). (*n* = 3 replica per surface).

Figure 7 shows proliferation ability of cells both by Actin cytoskeleton staining (Figure 8a) and quantitatively by measuring metabolic activity (Figure 8b). The first obvious data correspond to the different proliferation kinetic in control conditions of the two cell models tested. Note that independently to the tested surface, data in the control surface (Figure 8b) indicate just double metabolic activity in hMSCs grown 7 days compared to day 1, while C3H10T1/2 cells showed quadruple metabolic activity at 7 days compared to day 1. Those data related to differences in proliferation of the selected cell models were expected, and at the same time wanted, as they allowed us to test the effect of Mg and HA on cells with different properties.

Regarding tested surfaces, both qualitative imaging and quantitative data indicate good cell proliferation in all cases, which was at least comparable to the proliferation at the control surface. On the contrary, a positive effect of Mg was observed, with significantly increased proliferation in all cells seeded in Mg carrier membranes, in line with the bibliography suggesting magnetic ions as Mg enhances mesenchymal stem cell proliferation [57]. Note that PLA-Mg membranes surface should show some degradation at that time point (See weight loss of PLA-5 Mg/30 HA in Figure 3), but it seems that feature did not affect cells on that surface.

Further on, we tested the stability of confluent cell monolayer on all PLA-derived surfaces at longer term and observed cell monolayer detachment between day 8 and 10 days after cell seeding (Supplementary Figure S4). This behavior was observed in all PLA samples, including PLA control, and as a hypothesis this behavior could be related to local PLA biodegradation promoted by the cell-material interaction, which may promote a local pH change.

We then conducted osteogenic differentiation studies to define the role of HA and Mg in this process. As mentioned in the introduction, both Mg^2+^ and Ca^2+^ have been reported as osteoinductive ions [13], and therefore we pointed GBR as the main applicability of these membranes. Assays were conducted 7 days after seeding, and therefore we tested the expression of the early osteogenic marker alkaline phosphatase (ALP) (Figure 8). In all cases data ALP were normalized to cell proliferation, as we had previously observed there were differences in proliferation related to different growing surfaces. First, cells were grown in normal cell culture conditions (Figure 8a) and this approach showed that none of the composites induced ALP expression in C3H10T1/2 cells. However, testing on hMSCs showed increased ALP expression, indicating some osteoinductive activity of Ca^2+^ and Mg^2+^ on hMSCs, a feature which was previously reported [58].

Cells were tested again, but in this case using osteogenic differentiation media (Figure 8b), aiming to define any synergy or potentiation of osteogenic signals by Mg^2+^ and Ca^2+^ ions. In this case, data obtained from both C3H10T1/2 and hMSC cell cultures indicate that the presence of Ca^2+^ and Mg^2+^ increased the ALP expression of tested cells, suggesting they potentiate osteogenic signals. We observed a statistically significant synergic effect of Ca^2+^ and Mg^2+^ in inducing ALP in C3H10T1/2 cells, while this synergy was a trend for hMSCs. In line with our data, previous reports have established in vitro that supplementation of osteogenic media with Mg promotes differentiation on MSCs [59], while Ca^2+^ has been also reported as required for osteogenic differentiation [60].

## 4. Conclusions

**Figure 9:**
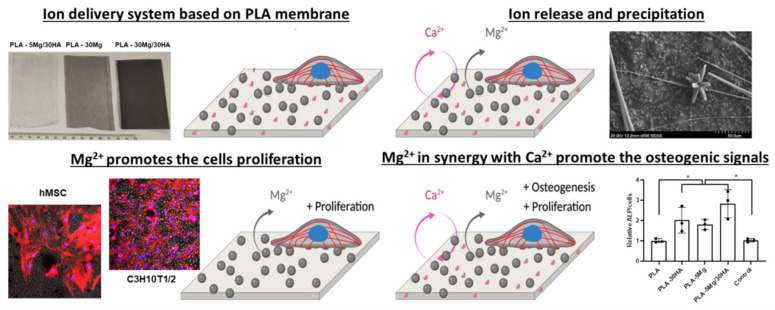
**Summary figure of proposed approach.** Tape casted methods used with PLA allow the formation of membranes potentially useful for Guided Bone Regeneration purposes. The method allows the loading of bioactive particles at PLA by colloidal suspension. This loading affects some parameters at material surface but did not affect materials biocompatibility. At in vitro cell culture studies, Mg loaded PLA membranes show improved cell proliferation, while Mg and Ca loaded PLA membranes show improved osteoinduction and proliferation, suggesting synergic effects taking place.

PLA membranes are used clinically in GBR approaches, and Mg^2+^ and Ca^2+^ have been reported as osteogenic ions, so we hypothesized that the addition of Mg^2+^ and Ca^2+^ to PLA membranes may provide useful osteogenic features to this clinical material (Figure 9). Therefore, this study aimed to define the bioactivity of PLA membranes fabricated using a colloidal suspension method, which allows the loading of high amounts of Mg^2+^ and Ca^2+^ carrier species in the PLA structure. The surface of these membranes shows the presence of Mg particles of 50 to 120 µm and HA particles of 1 to 3 um. The testing carried out in watertight conditions to simulate and mimic the in vitro cell culture test settings showed differences in Mg^2+^ and Ca^2+^ ions delivery and precipitation processes, including Mg(OH)_2_ flowerlike-crystal formation on the surface of membranes loaded with 30 wt.% of Mg (PLA-30 Mg). However, data indicate that the presence of Mg and its reactivity with media is the main driving force of the surface hydrophilicity and the hydration of PLA membranes, leading to PLA degradation, a feature which was more evident in samples with high amounts of Mg. These features were evaluated in in vitro cell culture context, were all tested PLA composites and were permissive to Mesenchymal Stem Cell adhesion and growth. Interestingly, Mg carrier membranes promoted cell proliferation and Mg, alone or in synergy with Ca and osteogenic signals, increased the expression of the ALP marker after 7 days of culture. Longer term assays indicate cell detachment, presumably related to cell-PLA interaction and biodegradation. Altogether, data indicate that the incorporation of Mg and HA to PLA membranes affects multiple physical parameters of the PLA membranes, including at surface topography. These, linked to the surface local Mg^2+^ and Ca^2+^ release accessible to the adhered cell, provide synergistic effects to promote proliferative and osteogenic features potentially useful for GBR purposes.

## Figures and Tables

**Table 1:** Membrane compositions (nominal values).

Membrane	PLA (wt.%)	Mg (wt.%)	HA (wt.%)
PLA	100	-	-
PLA-5 Mg	95	5	-
PLA-10 Mg	90	10	-
PLA-30 Mg	70	30	-
PLA-10 HA	90	-	10
PLA-30 HA	70	-	30
PLA-50 HA	50	-	50
PLA-5 Mg/30 HA	65	5	30
PLA-30 Mg/30 HA	40	30	30

**Table 2:** **Membrane surface properties**: surface average roughness (S_a_), average height to the peaks (R_h_), Kurtosis parameter (S_ku_), average contact angle (ϑ).

Membrane	S_a_ (µm)	R_h_ (µm)	S_ku_	ϑ (°)
PLA	-	-	-	83.32 ± 5.34
PLA-5 Mg	1.5	4.1	0.1109	84.4 ± 3.92
PLA-30 HA	1.1	3.3	0.0498	82.15 ± 7.37
PLA-5 Mg/30 HA	2.3	5.9	0.0393	80.48 ± 9.03

## Data Availability

Not applicable.

## Supplementary Materials

The following supporting information can be downloaded at: https://www.mdpi.com/article/10.3390/polym14122422/s1, Figure S1: SEM images of the different films; Figure S2: Evolution of Mg^2+^ (a) and Ca^2+^ ions release (b) in absolute values; Figure S3: Cell-seeding approach; Figure S4: Long-term cell cultures; Video S1: Mechanical properties of different PLA.

## Abbreviations

PLA, Polylactic acid 2; Mg, magnesium; HA, hydroxyapatite; Ca, calcium; PBS, Phosphate-buffered saline solution; ALP, Alkaline Phosphatase; MSC, Mesenchymal Stem Cells; GBR, Guided Bone Regeneration; TCP, tricalcium phosphate, THF, Tetrahydrofuran; PEI, Polyethylenimine; TMAH, Tetramethylammonium hydroxide; Sa, superficial average roughness; Rh, average height to the peaks; Sku, kurtosis parameter; ϑ, contact angle; SEM, scanning electron microscopy; EDS, energy dispersive X-ray spectroscopy; ICP-OES, Inductively coupled plasma–optical emission spectroscopy; DSA, Drop Shape Analyzer; C3H/10T1/2, mouse embryonic Mesenchymal Stem Cell; BME, Basal Medium Eagle; αMEM, MEM Alpha; hMSC, human bone marrow-derived Mesenchymal Stem Cell; MSCBM, Mesenchymal Stem Cell Basal Medium; UV, ultraviolet; DAPI, 4′,6-diamidino-2-phenylindole; NA, numerical aperture; Mg(OH)_2_, Magnesium hydroxide.
