# Supplementary material for: The Effect of Ca2+ and Mg2+ Ions Loaded at Degradable PLA Membranes on the Proliferation and Osteoinduction of MSCs
Source: Polymers (Basel). 2022 Jun 15;14(12):2422. doi: 10.3390/polym14122422 (PMC9228138; doi:10.3390/polym14122422)
Supplement: Supplementary file 1 [file polymers-14-02422-s001.zip › polymers-1763368-supplementary.pdf]

SUPPLEMENTARY MATERIAL

The Effect of Ca^{2+} and Mg^{2+} Ions Loaded at Degradable PLA Membranes on the Proliferation and Osteoinduction of MSCs

Sugoi Retegi-Carrión [1], Ana Ferrandez-Montero [2,3,*], Alvaro Eguiluz [2], Begoña Ferrari [2,*] and Ander Abarrategi [1,4,*]

[1] Center for Cooperative Research in Biomaterials (CIC biomaGUNE), Basque Research and Technology Alliance (BRTA), 20014 Donostia-San Sebastian, Spain; sretegi@cicbiomagune.es
[2] Institute of Ceramic and Glass, Spanish National Research Council (CSIC), 28049 Madrid, Spain; alvaro.eguiluz@icv.csic.es
[3] Laboratoire de Physicochimie des Polymères et des Interfaces (LPPI), I-Mat, CY Cergy Paris Université, CEDEX, 95000 Neuville sur Oise, France
[4] Ikerbasque, Basque Foundation for Science, 48009 Bilbao, Spain
[*] Correspondence: aferrandez@icv.csic.es (A.F.-M.); bferrari@icv.csic.es (B.F.); aabarrategi@cicbiomagune.es (A.A.)

SUPPLEMENTARY MATERIAL LIST

Supplementary Figure S1. SEM images of the different films.

Supplementary Video S1. Mechanical properties of different PLA. Video provided in a separate file.

Supplementary Figure S2. Evolution of Mg^{2+} (a) and Ca^{2+} ions release (b) in absolute values.

Supplementary Figure S3. Cell-seeding approach.

Supplementary Figure S4. Long-term cell cultures.

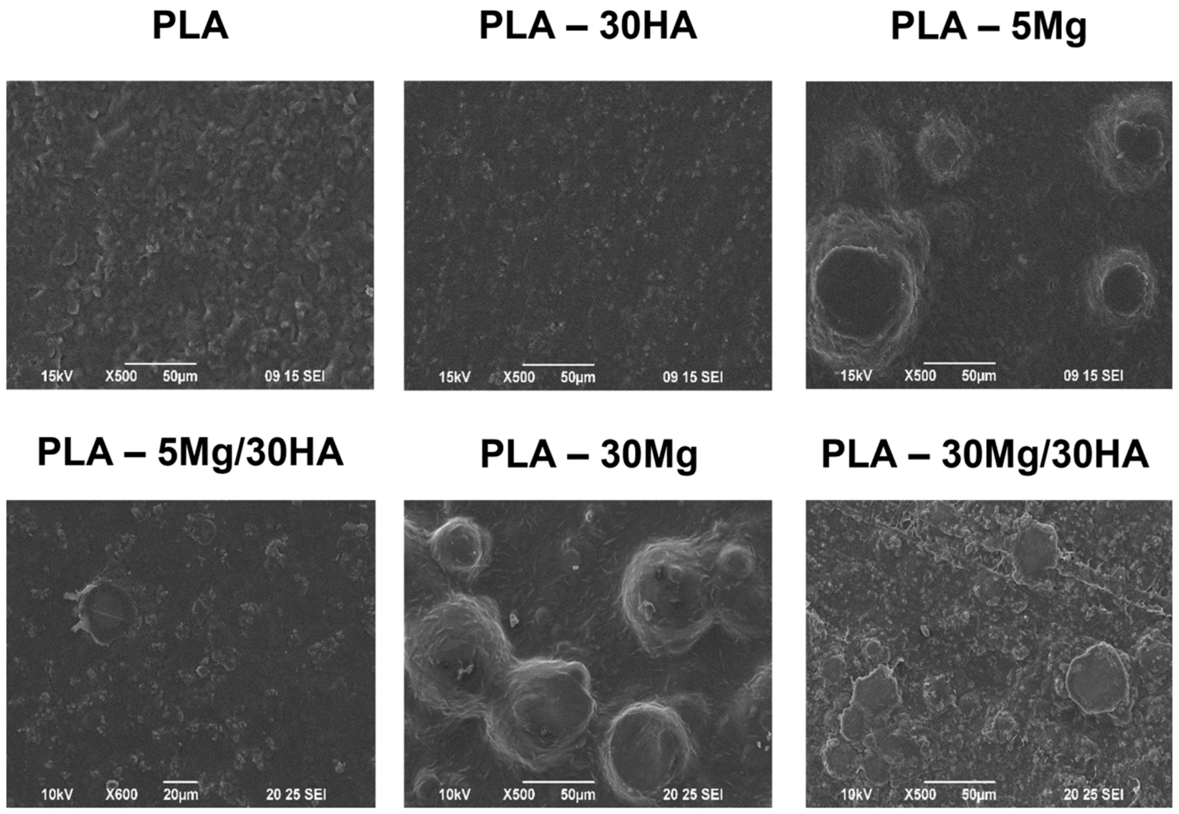

Supplementary Figure S1. SEM images of the surface of the different films.

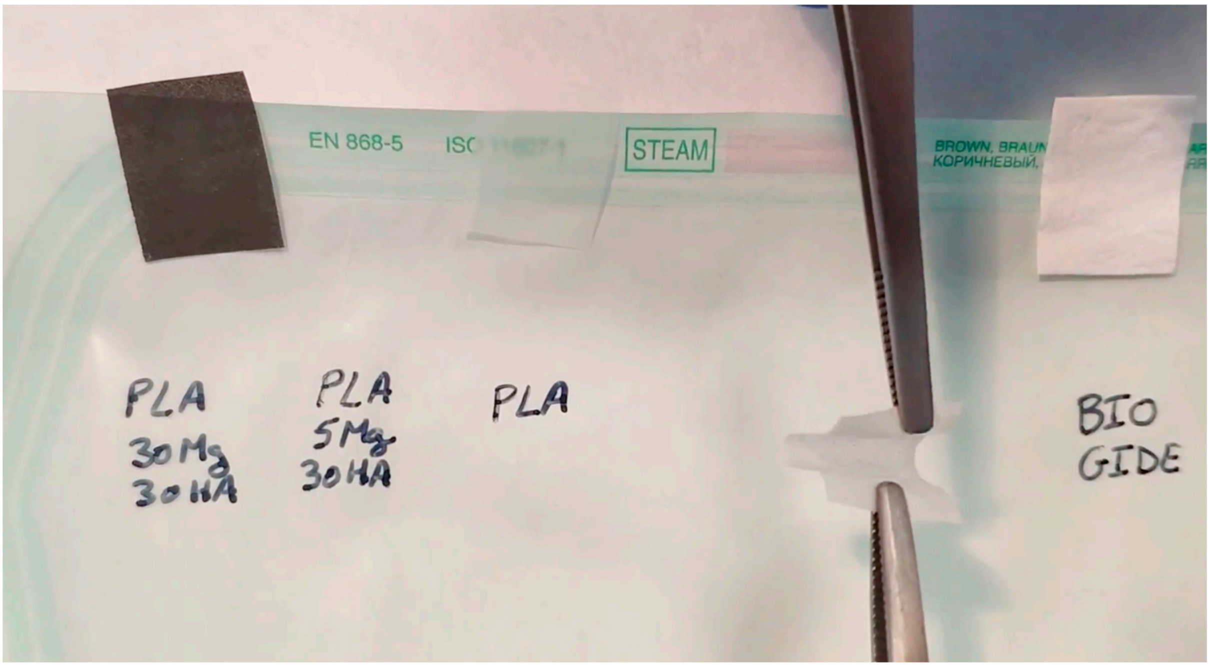

Supplementary Video S1. Mechanical properties of different PLA. Video provided in a separate file.

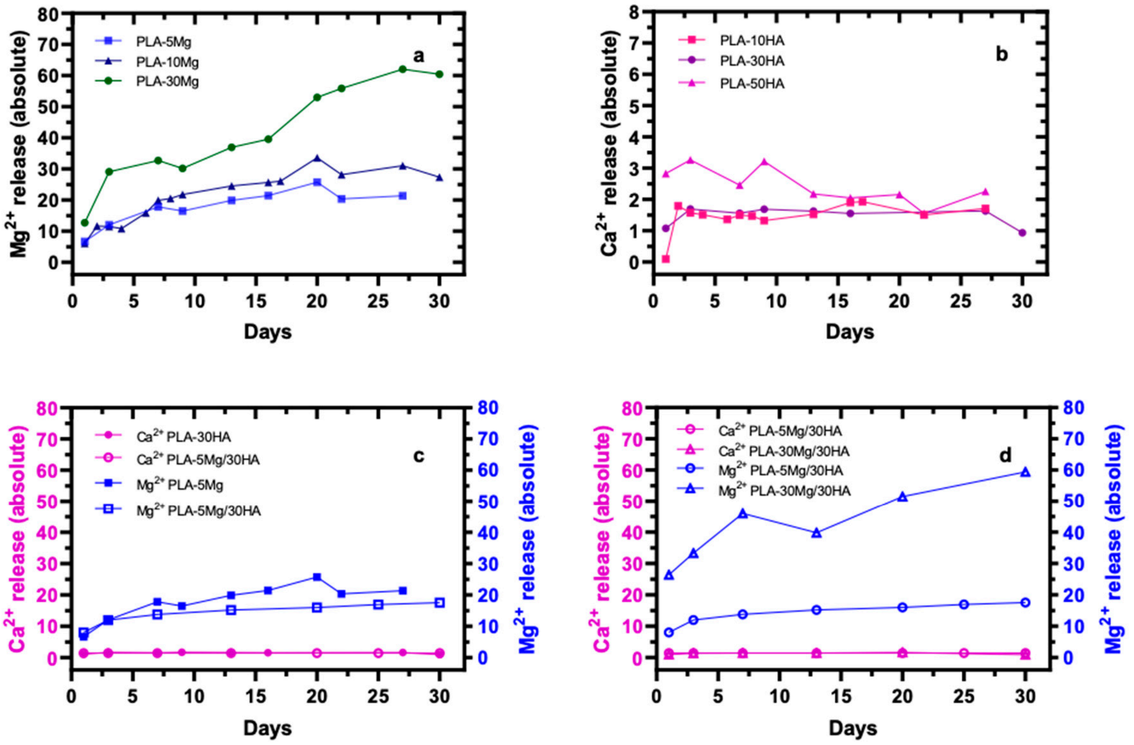

Supplementary Figure S2. Evolution of Mg^{2+} (a) and Ca^{2+} ions release (b) in absolute values.

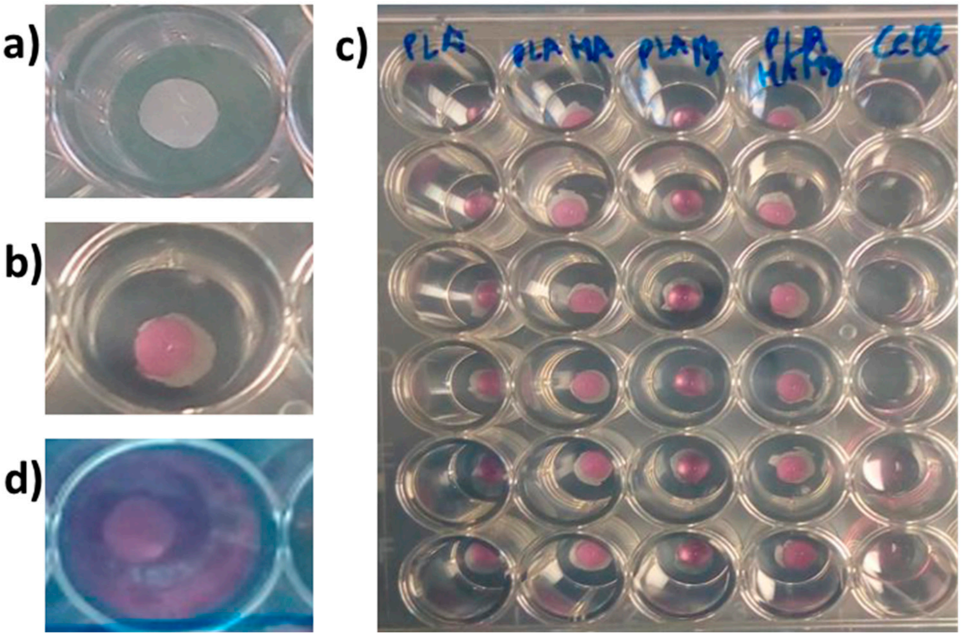

Supplementary Figure S3. Cell-seeding approach. (a) PLA-based films were cut in disks and deposited in cell-culture multiwell plates. (b) A drop of cell culture media with cells was seeded on top of each film disk. (c) An example of a typical cell culture assay plate with multiple films seeded. Cell culture glass coverslips with similar diameter to PLA-based films were deposited in control wells and similar cell-seeding approach was conducted on them. Cells were allowed to attach for 4 hours at cell culture conditions. (d) Cell culture media was added carefully to each well, ensuring PLA-based films remained in the bottom of the well.

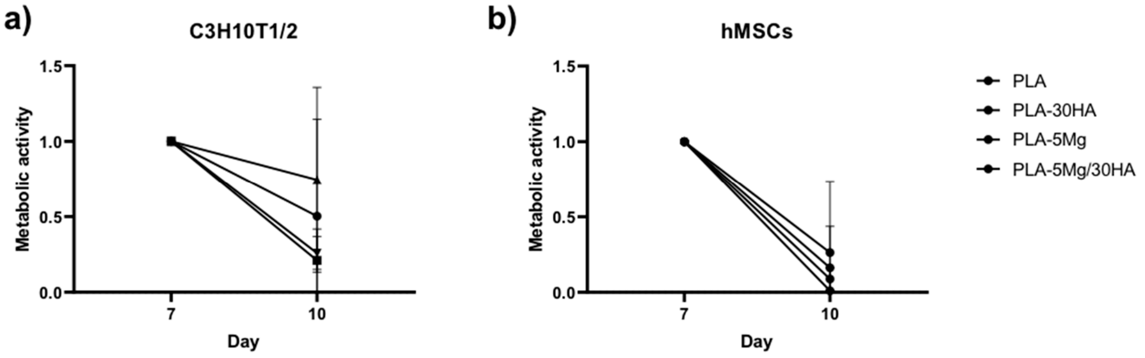

Supplementary Figure S4. Long-term cell cultures. In preliminary studies (a) C3H10T1/2 and (b) hMSC cells were tested at long term at different time points, aiming to define if film degradation during cell culture affects cell viability. We observed cells detach from all PLA films at time points longer that 7-10 days. Data are provided normalized to metabolic activity of cells grown at each surface at day 7.
